# A Case of High-Dose-Rate Brachytherapy Under Endoscopic Retrograde Cholangiopancreatography (ERCP) for Intraductal Papillary Neoplasm of the Bile Duct

**DOI:** 10.7759/cureus.58254

**Published:** 2024-04-14

**Authors:** Masumi Kawaguchi, Tomohiro Itonaga, Ryuji Mikami, Aoi Sukeda, Takayoshi Tsuchiya, Atsushi Sofuni, Takao Itoi, Kazuhiro Saito

**Affiliations:** 1 Department of Radiology, Tokyo Medical University Hospital, Tokyo, JPN; 2 Department of Anatomic Pathology, Tokyo Medical University Hospital, Tokyo, JPN; 3 Department of Gastroenterology and Hepatology, Tokyo Medical University Hospital, Tokyo, JPN

**Keywords:** high-dose-rate (hdr) brachytherapy, peroral cholangioscopy, radiotherapy (rt), ercp, ipnb

## Abstract

Intraductal papillary neoplasm of the bile duct (IPNB) represents a relatively nascent pathological entity, recognized as a precancerous condition within the spectrum of cholangiocarcinoma. Surgical intervention is advocated for all patients with IPNB due to their susceptibility toward obstructive jaundice, cholangitis, and the heightened likelihood of malignant transformation. Nonetheless, the efficacy of radiation therapy for IPNB cases that are either inoperable or refractory remains inadequately substantiated. Herein, we present a case study of an IPNB patient who declined surgery, and a commendable local control was accomplished solely through the implementation of brachytherapy utilizing Ir-192. A septuagenarian Japanese man presented at our medical institution with the chief complaint of jaundice and was subsequently diagnosed with IPNB. The IPNB lesion extensively spanned from the lower intrapancreatic bile duct to the right (extending to B5/B8) and left bile ducts (up to just before B4). The patient underwent weekly endoscopic retrograde cholangiopancreatography (ERCP) sessions. The prescribed treatment regimen encompassed 36 Gy/6 Fr high-dose-rate brachytherapy (HDR-BT) administered once per week during ERCP, with each treatment session adhering to a timeframe not exceeding two hours. Two months following the initiation of treatment, a biliary endoscopy demonstrated complete resolution of the tumor lesion and amelioration of jaundice. The only observed acute adverse event was grade 2 hepatic dysfunctions. To the best of our knowledge, this represents the first documented instance of HDR-BT employed in IPNB management, suggesting its potential as a viable alternative for inoperable or refractory IPNB cases.

## Introduction

Intraductal papillary neoplasm of the bile duct (IPNB) represents a rare neoplastic entity that emerges from the epithelial lining of both intra- and extrahepatic bile ducts, serving as a precursor lesion to invasive cholangiocarcinoma [1]. IPNB demonstrates a proclivity for inducing obstructive jaundice and cholangitis, and a substantial proportion (40-74%) of cases manifests malignancy upon initial diagnosis, thereby necessitating surgical intervention in all instances [2]. A report from Korea showed a five-year overall survival rate of 80.9% for patients with surgically resectable IPNB. An observational study using the Surveillance, Epidemiology, and End Results (SEER) database reported a poor prognosis in patients who refused or were unable to undergo surgery and that adjuvant chemotherapy improved prognosis [3]. 

There have been published case reports documenting the utilization of external beam radiation therapy (EBRT) for the management of IPNB [4]. However, to the best of our knowledge, there are no reported cases of high-dose-rate brachytherapy (HDR-BT) in the treatment of IPNB. It is suggested that for cholangiocarcinoma, HDR-BT may be associated with a favorable overall survival [5]. The advantage of HDR-BT is that, in contrast to EBRT, it does not require respiratory motion management during irradiation and allows dose reduction to the liver. In this report, we present a case in which HDR-BT was performed under endoscopic retrograde cholangiopancreatography (ERCP) and good local control was achieved.

## Case presentation

**Figure 1:**
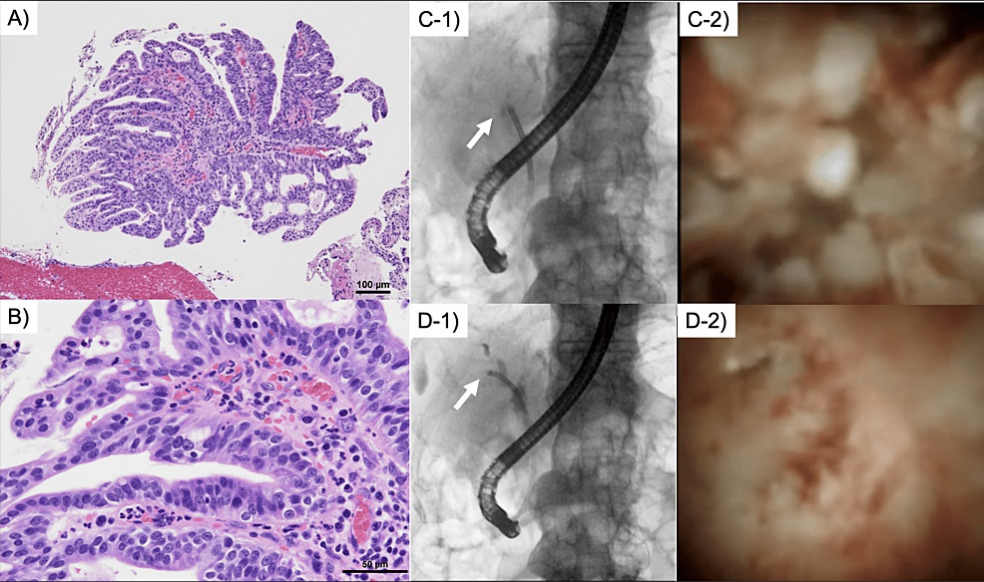
Findings of hyperplasia of neoplastic epithelium exhibiting papillary/villous structures within the intrahepatic bile duct lumen. A) At 100× magnification with hematoxylin–eosin staining, B) at 400×. C-1) and D-1) show abdominal X-ray images showing the location of peroral cholangioscopy (PCOS), and C-2) and D-2) show images of  PCOS; C) is located at the hilar bile duct (white arrow), D) at the origin of the right bile duct (white arrow), showing white papillary elevations with mucin production in the bile duct.

The patient is a 74-year-old man with no past medical history, who presented with general malaise. The blood test from his previous hospital revealed liver dysfunction, and based on the results of imaging and biopsy, he was referred to our hospital for treatment for suspected IPNB. The patient provided written consent for the use of imaging data for research involving his examinations at our hospital. An initial peroral cholangioscopy (POCS) showed that the lesion extended from the porta hepatis to just above the papilla, to B5 and B8 on the right side, and to just before B4 on the left (Figure 1). An endoscopic biopsy revealed high-grade dysplasia from the hilar region and low-grade dysplasia from B4 and B2/3 and intrapancreatic bile duct. The computed tomography (CT) and magnetic resonance imaging (MRI) scans revealed the existence of a neoplasm characterized by the expansion of the intrahepatic bile ducts derived from the common bile duct, accompanied by delayed enhancement within the luminal space of the bile duct. No evidence of lymph node metastasis or distant metastasis was detected.

In the surgical evaluation, it was determined that in order to pursue a curative intent, an extended right hepatectomy combined with pancreaticoduodenectomy was deemed necessary. Preoperative examinations revealed a mild elevation of the indocyanine green retention rate at 15 min, with a value of 15, and the CT volumetry analysis demonstrated a resection rate of 42.6% for right hepatectomy and 30.3% for right trisectionectomy. The patient deemed the surgical risk to be high and opted for radiation therapy. Considering the effects of hepatic function caused by external beam radiation therapy, we opted for the selection of HDR-BT treatment under ERCP guidance. The regimen of radiation therapy was established as a weekly treatment, delivering a dose of 36 Gy/6 Fr. Adjusting to the spread of the tumor, a total of four fractions were designated for the common bile duct-right intrahepatic bile duct, while two fractions were allocated for the common bile duct-left intrahepatic bile duct.

**Figure 2:**
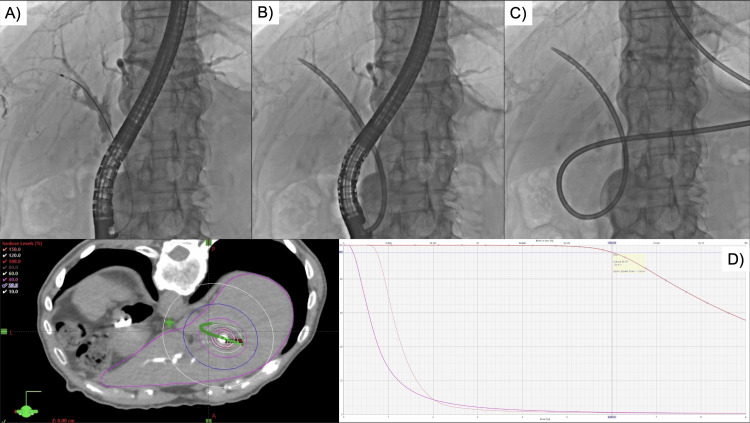
High-dose-rate brachytherapy under endoscopic retrograde cholangiopancreatography for Intraductal papillary neoplasm of the common bile duct-right intrahepatic bile duct A) Performing endoscopic retrograde cholgiopancreaticography (ERCP) and confirming lesion with cholangiography (Step 1). B) 10 Fr ENBD tube implanted via guidewire (Step 2). C) Confirmation that the source can pass through the brachytherapy applicator (Step 3). D) Treatment planning computed tomography (CT) is performed in the prone position to create a treatment plan (Step 4). The dose-volume histogram for the initial treatment plan shows 6.5 Gy for 90% of Target (red), 3.0 Gy for D1cc in the duodenum (pink), and a normal mean liver dose (blue) of 1.0 Gy.

The HDR-BT procedure consisted of five steps (Figure 2). First, the gastroenterologist performed ERCP under sedation to visualize the lesion using cholangiography. Second, a 10 Fr endoscopic nasociliary drainage (ENBD) tube was placed in the lesion and a 4.7 Fr brachytherapy applicator was placed inside. The HDR-BT treatment system cannot supply the radioactive source beyond a distance of up to 150 cm. Therefore, the insertion range of the ENBD tube is limited to less than 150 cm. Third, after confirming that the source could pass through the applicator, a treatment planning CT was performed in the prone position, and the treatment plan was created by VariSeed (Varian Medical Systems, Palo Alto, CA, USA). When creating the treatment plan, the target was defined as the 5-mm outer-wall margin of the ENBD tube, and the treatment plan was created so that 6 Gy was prescribed for 90% of the target volume. The organs at risk (OARs) were set to be the duodenum and liver around the tumor. Dose constraints were adjusted so that the D1cc of the duodenum was less than the prescribed dose (6 Gy) and the mean liver dose (MLD) was less than 20 Gy. Fourth, the Ir-192 high-dose-rate remote after the loading system (VariSourceTM; Varian Medical Systems) was used to administer treatment. The treatment time was approximately 20 minutes in the prone position. Fifth, after completion of the treatment, the applicator was removed, and an ENBD tube was placed for drainage. Each treatment session typically lasted 1.5 to two hours. The total doses for the OAR were MLD = 5.1 Gy and D700cc = 2.0 Gy for the liver and D2cc = 14.3 Gy and D1cc = 16.0 Gy for the duodenum.

Radiotherapy was completed as scheduled with no grade ≥3 adverse events during the six-week treatment (Table 1). Pre- and post-treatment, the Child-Pugh score remained the same at A, but the point changed from 5 to 6 because of the decrease in albumin. The general malaise observed pre-treatment did not change post-treatment.

**Table 1:** Laboratory findings pre- and post-treatment

	Pre-treatment	Post-treatment
Aspartate transaminase	56 U/L	92 U/L
Alanine transaminase	63 U/L	88 U/L
Total bilirubin	0.51 mg/dL	0.54 mg/dL
Direct bilirubin	0.25 mg/dL	0.18 mg/dL
Alkaline Phosphatase	206 U/L	552 U/L
γ-Glutamyl Transpeptidase	205 U/L	685 U/L
Albumin	4.1 g/dL	3.4 g/dL
CA19-9	42.7 U/mL	68.7 U/mL
CEA	< 2.0 ng/mL	< 2.0 ng/mL

**Figure 3:**
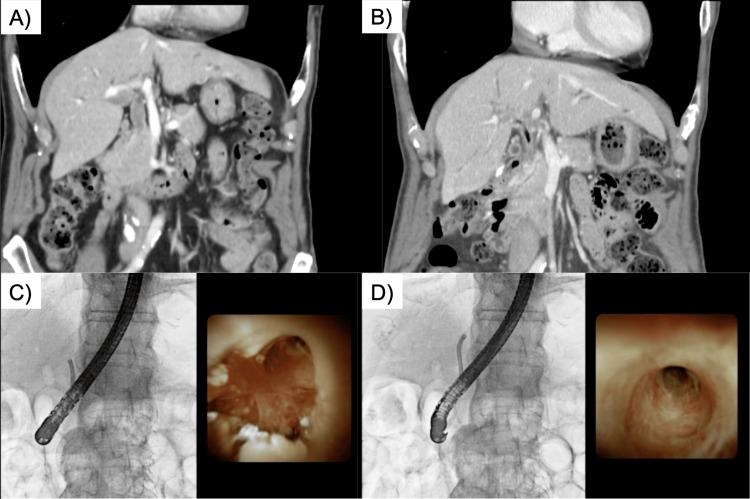
Comparison of CT and oral cholangioscopic images pre- and post-high-dose-rate brachytherapy A) Represents the pretreatment coronal image of contrast-enhanced computed tomography (CT), while B) represents the post-treatment coronal image of contrast-enhanced CT. The solid component in the bile duct that was showing a contrast effect before treatment is obscured. C) and D) represent the post-treatment evaluation images obtained using peroral cholangioscopy (POCS). C) focuses on the hepatic hilum, while D) captures the left bile duct. The papillary lesion observed prior to treatment demonstrates improvement. A biopsy was performed on the suspicious area suggestive of residual disease, revealing evidence of inflammation.

Two months after completion of treatment, POCS and contrast-enhanced CT were performed for evaluation (Figure 3).

In POCS, a slight papillary elevation was observed at the B2/3 branching site, and scar-like changes were identified from the papilla of Vater to the bilateral bile duct origins. Although a biopsy was performed in the same area, only inflammatory changes were evident, and no definitive tumor was identified. Contrast-enhanced CT obscured the tumor lesions within the bile ducts, and no obvious metastatic findings could be noted. Residual disease was suspected in the right lobe of the liver, and a resection of the right lobe of the liver was proposed; however, the patient requested a follow-up procedure. The patient was admitted to another hospital due to jaundice 11 months after the start of HDR-BT. A close examination revealed tumor recurrence in the left branch of the bile duct, and a stent was placed. At 21 months after post-treatment, there were no findings other than recurrence from the left branch of the bile duct and no serious HDR-BT-related adverse events.

## Discussion

Previous studies have reported that IPNB is most common in intrahepatic or hilar regions and can be divided radiologically into four subtypes based on the size and morphology of the intraductal mass, degree of mucin secretion, and location of the tumor [6]. The four types are as follows: 1) masses with proximal ductal dilatation, 2) disproportionate dilatation without masses, 3) masses with proximal and distal ductal dilatation, and 4) cystic lesions. This case corresponded to Type 3, which is the most common type among IPNB cases. An important aspect of the treatment of IPNB is the precise identification of the tumor progression. While CT is commonly used, its sensitivity is limited to around 50%, whereas MRI, including MRCP, demonstrates the highest sensitivity (65.5%) among the imaging modalities [6,7]. However, in the case of IPNB, the mucin present in the neoplasm resembles the mucous constituents of bile, rendering it challenging to precisely quantify the extent of disease progression through radiological assessments [8]. Conversely, POCS has been reported to possess the ability to identify lesions in approximately 30% of cases when the tumor remains undetected on radiological imaging [7]. In this case, the exact location of tumor extension could not be determined by contrast-enhanced CT alone, and POCS was performed to determine that the tumor extended to the lower common bile duct. An analysis of patients who underwent surgery for IPNB reported a significantly lower overall survival for patients with extrahepatic extension of the tumor compared to those with intrahepatic localization of the tumor, suggesting that POCS plays an essential role in preoperative diagnosis [9].

The mainstay of treatment for IPNB is surgery, and the role of radiation therapy remains controversial. A study that analyzed the SEER database revealed that 60.1% of patients underwent surgery, while only 17.5% opted for radiation therapy [3]. Furthermore, this study did not find any statistically significant differences in the overall and cancer-specific survivals with regard to radiation therapy in patients with IPNB. One limitation of the database study was that the radiation therapy group included postoperative and palliative radiotherapy, making the role of definitive radiotherapy unclear. There have been only a few case reports demonstrating the efficacy of radiotherapy for IPNB, and to our knowledge, this is the first case of HDR-BT for local control. While treating this case, we used HDR-BT for cholangiocarcinoma. This treatment was based on HDR-BT for unresectable cholangiocarcinoma. HDR-BT offers the advantage of delivering high doses of radiation to the lesion while reducing the dose to surrounding normal tissues, as compared to conventional EBRT. The approach to the tumor is either ERCP or percutaneous transhepatic cholangiodrainage (PTCD), the latter being more common. Although ERCP is a more difficult procedure than PTCD, it has the advantages of being minimally invasive, carries a low risk of seeding, and can easily identify the lesion by cholangiography.

The fractionation schedule in this case was determined based on considerations of safety and efficacy. Data regarding the application of HDR-BT as a monotherapy curative modality for the treatment of cholangiocarcinoma are scarce. Mattiucci et al. reported good safety and a one-year overall survival rate of 59% with a dose prescription of 25 Gy/5 Fr in a phase I study using HDR-BT [10]. In a phase I trial of stereotactic body, radiotherapy for unresectable hepatocellular carcinoma and cholangiocarcinoma, delivered 36 Gy in six fractions, resulted in grade 3 liver dysfunction in 12% of patients, and the median survival of cholangiocarcinoma was reported to be 15 months [11]. A meta-analysis of radical EBRT for inoperable cholangiocarcinoma reported that median biologic equivalent doses of 80.5 Gy or higher were associated with better local control rates and improved overall survival rates [12]. With our dose fractionation (36 Gy/6 Fr), the biological equivalent doses are low at 57.6 Gy, but a simple evaluation is difficult due to the differences between brachytherapy and EBRT. The results of HDR-BT monotherapy (25 Gy/5 fr, BED = 37.5 Gy) for cholangiocarcinoma by Mattiucci et al. are consistent with the fact that four HDR-BT sessions (24 Gy/4 fr, BED = 38.4 Gy) were sufficient for tumor control in the right intrahepatic bile duct. In this case, the site of recurrence was the left intrahepatic bile duct within the irradiation field, a site where HDR-BT had only been performed twice. Therefore, we consider that the cause of the recurrence was under-prescribed dose rather than under-irradiated filed. The most common adverse event in radiation therapy for cholangiocarcinoma is gastrointestinal complications [13]. There are reports suggesting that late small-bowel obstruction or perforation events are observed in 2-9% of patients when the total dose exceeds 50 Gy in an equivalent dose in 2 Gy fractions (EQD2) [14]. In our treatment protocol, the EQD2 was 48 Gy, ensuring safety in the dose-fractionation scheme employed at our institution. In this treatment, a three-dimensional image-guided small source therapy was used to accurately assess the dose to the tumor and OAR. The 5-mm submucosal dose reference point, which was commonly used in two-dimensional treatment, was applied to the target volume of 5 mm from the outer wall of the ENBD tube, and the prescribed dose was planned to be delivered to 90% of the target volume [15,16].

The challenge in the ERCP-guided HDR-BT procedure in this case lay in the inability to simultaneously insert the applicator tubes on both sides due to the diameter restrictions of the ENBD tube, despite the lesions extending from the common bile duct to the left and right intrahepatic bile ducts. One potential solution to this issue was the addition of an applicator under PTCD guidance for the contralateral intrahepatic bile duct, in addition to the applicator under ERCP guidance. In this treatment, we decided against adding PTCD considering the invasiveness and extent of the lesion. However, it can be considered as an option in future cases.

## Conclusions

In this study, HDR-BT under ERCP was performed for IPNB refusal of surgery, and the treatment was completed without severe adverse events. The range of IPNB extension is difficult to determine by radiological imaging alone, and POCS should be performed prior to HDR-BT. To the best of our knowledge, this is the first case report of HDR-BT in the treatment of IPNB. A larger number of cases is needed to determine the optimal dose fractionation of HDR-BT for IPNB.
